# *Aquilegia* B gene homologs promote petaloidy of the sepals and maintenance of the C domain boundary

**DOI:** 10.1186/s13227-017-0085-7

**Published:** 2017-11-28

**Authors:** Bharti Sharma, Elena M. Kramer

**Affiliations:** 1 Department of Biological Sciences, California Polytechnic State University Pomona, 3801 West Temple Avenue, Pomona, CA 91768 USA; 2 Department of Organismic and Evolutionary Biology, Harvard University, 16 Divinity Ave., Cambridge, MA 02138 USA

**Keywords:** *Aquilegia*, Homeosis, Floral development, MADS box genes, ABC model, Petaloidy

## Abstract

The model *Aquilegia coerulea* x “Origami” possesses several interesting floral features, including petaloid sepals that are morphologically distinct from the true petals and a broad domain containing many whorls of stamens. We undertook the current study in an effort to understand the former trait, but additionally uncovered data that inform on the latter. The *Aquilegia* B gene homolog *AqPI* is shown to contribute to the production of anthocyanin in the first whorl sepals, although it has no major role in their morphology. Surprisingly, knockdown of *AqPI* in *Aquilegia coerulea* x “Origami” also reveals a role for the B class genes in maintaining the expression of the C gene homolog *AqAG1* in the outer whorls of stamens. These findings suggest that the transference of pollinator function to the first whorl sepals included a non-homeotic recruitment of the B class genes to promote aspects of petaloidy. They also confirm results in several other Ranunculales that have revealed an unexpected regulatory connection between the B and C class genes.

**Electronic supplementary material:**

The online version of this article (10.1186/s13227-017-0085-7) contains supplementary material, which is available to authorized users.

## Findings

### Background

Botanists make very clear distinctions between *petals* and *petaloidy*. Petals, being synonymous with the corolla, are defined by the Plant Ontology Consortium as the inner whorl of non-reproductive organs that surround the androecium [1]. While these organs are often showy, the primary traits that define them are their position in the flower (the second whorl) and the fact that they are sterile. In contrast, “petaloidy” refers to an organ’s appearance and indicates non-photosynthetic organs that are modified for pollinator attraction. Petaloid organs can occur in any whorl of the flower or even be extra-floral (e.g., bracts in *Poinsettia*). Both second whorl petals and the general appearance of petaloidy have evolved many different times independently across the angiosperms [2]. The discovery of the genetic program controlling floral organ identity, the so-called ABC model [3], gave us candidate genes—homologs of the B class petal identity genes *APETALA3* (*AP3*) and *PISTILLATA* (*PI*)—to explore the molecular basis of petaloidy in all its possible iterations.

The homeotic nature of the ABC model suggests a simple model in which petaloid organs can arise by spatial shifts of the B gene expression domain [4, 5]. This appears to be the case in many monocots with undifferentiated petaloid perianths, such as tulips or lilies, in which B gene expression is commonly observed in all of the perianth organs (reviewed [6]). Such undifferentiated perianths are less common in dicots, but a similar pattern has been described for lobelioid *Clermontia* [7]. In these examples, the first and second whorl organs are very similar at maturity, suggesting that a single organ identity program is being broadly expressed. In many other instances, however, the sepals may be petaloid, but they differ considerably in morphology relative to the petals. Several such examples have now been studied, and most of these petaloid sepals lack B gene expression, suggesting that the development of petaloid features in these organs is due to convergence rather than any degree of homeosis (e.g., [8–10]).

**Fig. 1:**
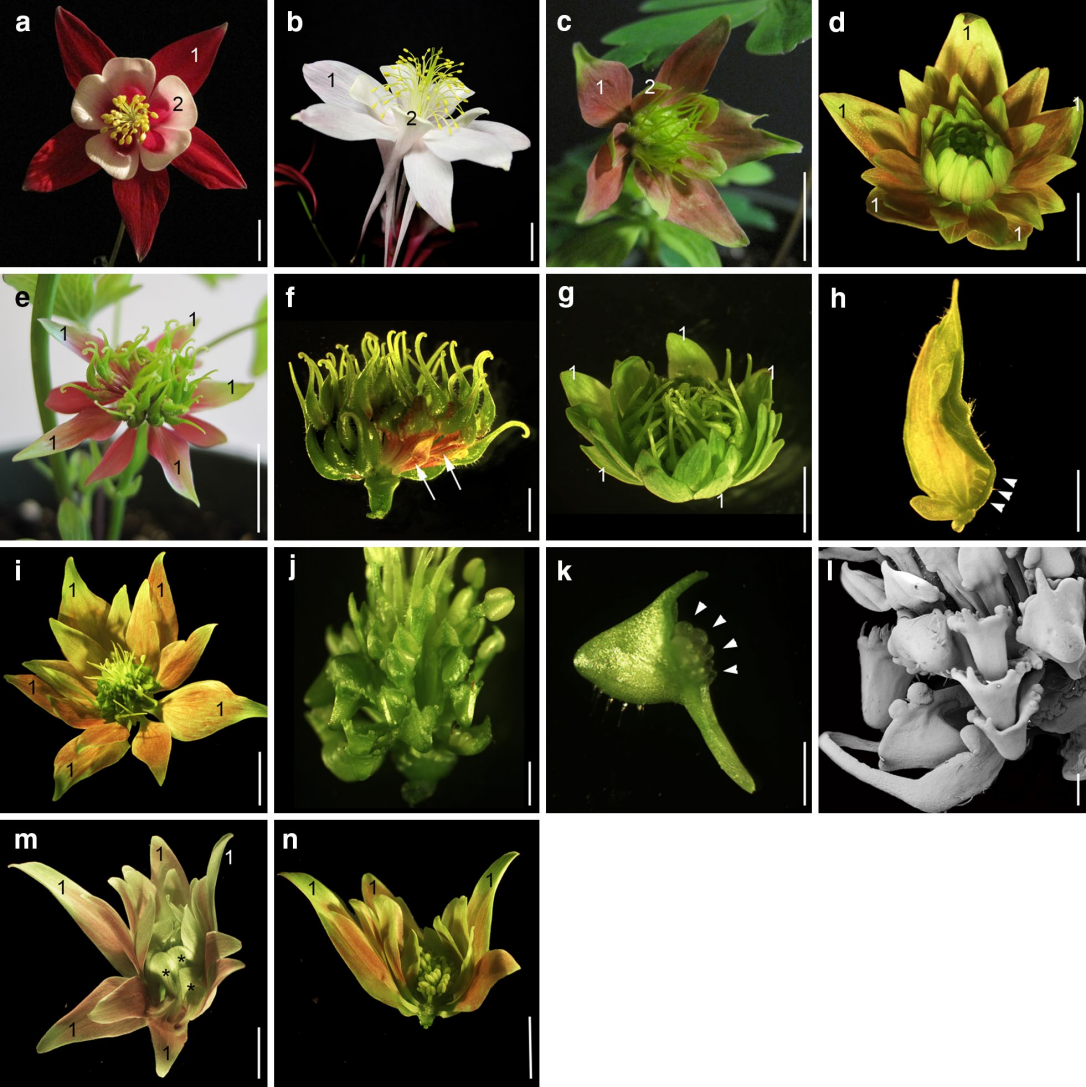
Phenotypes recovered in *Aquilegia x coerulea AqPI*-VIGS flowers. **a** Wild-type flower with labeled first whorl sepals (1) and second whorl petals (2). **b** Flower with only *AqANS*-silencing, sepals and petals labeled as in **a**. **c**–**g**, **i**, **m**, **n** Range of floral phenotypes recovered from *AqPI*-VIGS plants. Wherever possible, we have indicated the first whorl sepals with the label “1.” All other sepals are transformed petals or stamens. In most of these flowers, all sepals, regardless of their position, show varying degrees of color loss, ranging from pale pink (e.g., **c**, **d**) to pale green (e.g., **g**). While half of the recovered flowers exhibited the standard B class mutant phenotype with petal to sepal and stamen to carpel transformation (e.g., **c**), we commonly observed transformations in which the outer whorls of reproductive organs became sterilized, most often into sepals, rather than the expected stamen to carpel transformation (e.g., **d**, **g**, **i**, **m**, **n**). Weak or variable silencing produced complex organs, including sepal/carpels, as seen in the flower in **e**, which is shown with its perianth removed in **f**. Arrows indicate sepaloid base of chimeric carpels. **h** A single sepal/carpel chimeric organ from the flower in **g**, with ectopic ovules indicated by arrowheads. Also observed in inner whorls were petal/carpels, as shown in **i**–**l**, and sepal/petals, as in **m** (asterisks indicate inner petaloid organs). The reproductive whorls of the flower in **i** are shown under light microscopy in **j** and scanning electron microscopy in **l**. A single petal/carpel chimera is shown in **k**, with arrowheads indicating ectopic ovules. In flowers showing the weakest silencing, the petals and outermost stamens were transformed into sepals, but internal stamen whorls retained their identity (**n**). Size bars 10 mm in **a**–**e**, **g**, **i**, **m**, **n**; = 2.5 mm in **f**, **h**, **j**; = 1 mm in **k** and **l**

So are there any instances where B genes contribute to the petaloidy of the sepals? There is one clear example, in orchids, in which B class genes appear to be critical to the establishment of separate petaloid identity programs in both the sepals and petals via the deployment of *AP3* paralogs [11, 12]. In *Aquilegia*, previous work has shown that the B class genes do not contribute to the identity of the sepals, either in terms of their gross morphology or their cell types [13]. However, these functional tests were always done using the *ANTHOCYANIDIN SYNTHASE (AqANS)* gene as a marker (e.g., Fig. 1b), so it cannot be ruled out that the B gene homologs contribute to anthocyanin production. We, therefore, decided to repeat this experiment using a virus-induced gene silencing (VIGS) construct that only contained *AqPI* in order to determine whether color production in the sepals was affected. We have found that expression levels of multiple members of the anthocyanin pathway are reduced in these flowers, but, in addition, we recovered a novel phenotype, suggesting that the B class genes are required for the maintenance of *AGAMOUS* (*AG*) homolog expression in the outer whorls of stamens.

## Results

### *AqPI*-VIGS plants exhibit a range of floral phenotypes

**Fig. 2:**
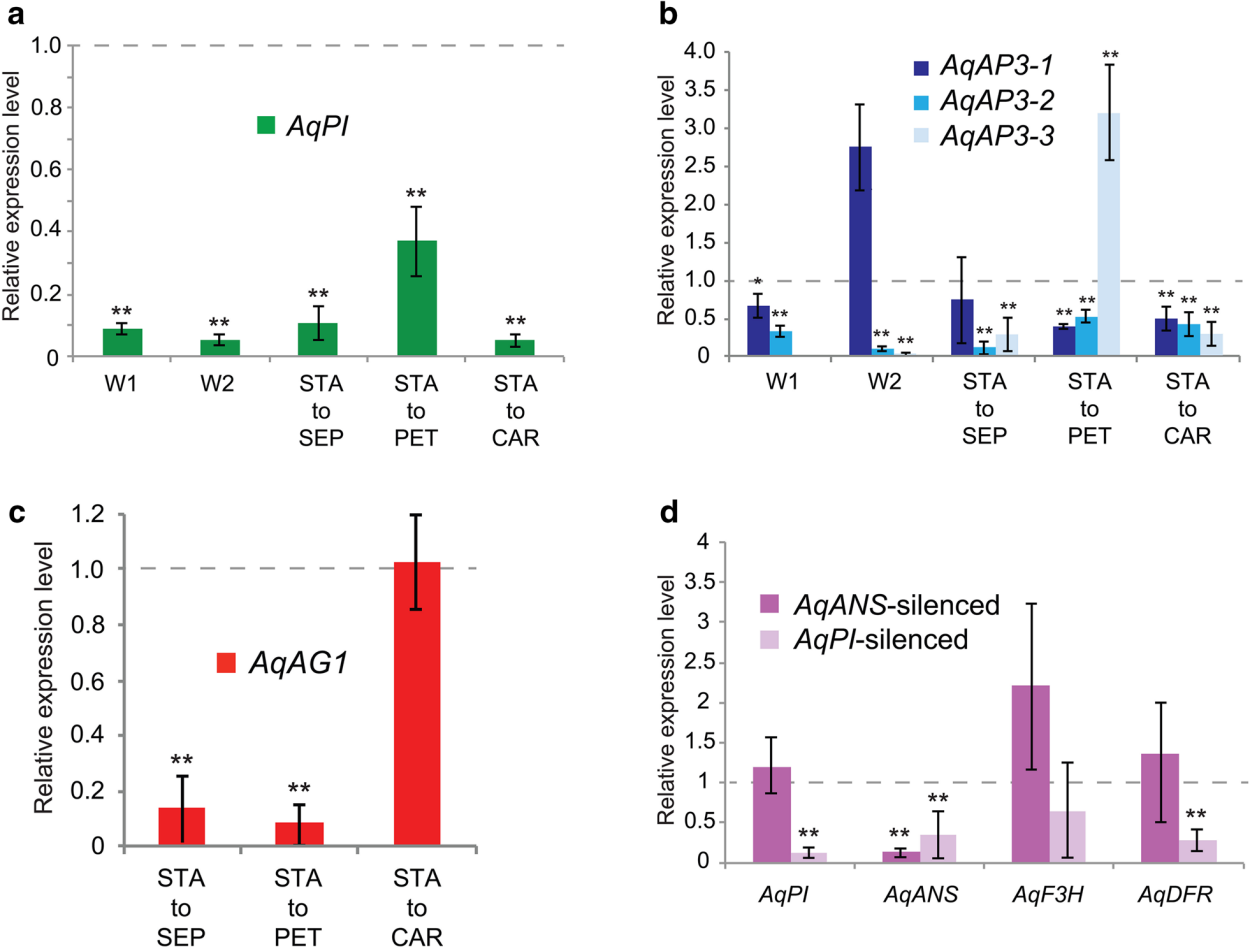
qRT-PCR analyses of gene expression levels in *AqPI*-VIGS tissue. **a** Expression of *AqPI* in first whorl organs (W1), second whorl organs (W2), stamens transformed into sepals (STA to SEP), stamens transformed into petals (STA to PET), and stamens transformed into carpels (STA to CAR). All values are normalized to the expression of *AqPI* in control *AqANS*-VIGS samples for each organ type (dotted line). **b** Expression of the three *Aquilegia AP3* homologs in same samples as **a**. All values are normalized to the expression levels of each gene in control samples for each organ type (dotted line). **c** Expression of *AqAG1* in stamens transformed to sepals (STA to SEP), stamens to petals (STA to PET), and stamens to carpels (STA to CAR). All values are normalized to the expression of *AqAG1* in control *AqANS*-VIGS stamens (dotted line). **d** Expression of *AqPI*, *AqANS*, *AqF3H*, and *AqDFR* across two classes of sepal tissue: *AqANS*-VIGS control tissue and *AqPI*-VIGS tissue. All values are normalized relative to the expression of each gene in untreated sepals (dotted line). All vertical axes represent relative expression levels, all asterisks indicate expression levels that are significantly different from the reference control organs, and all error bars represent standard deviation

After treating 90 plants with TRV2-*AqPI*, we recovered 50 flowers with homeotic phenotypes, which fell into two broad classes. In the first class, there were 25 flowers that displayed canonical B gene mutant phenotypes with petal to sepal and stamen to carpel transformations (Fig. 1c). *Aquilegia* has a fifth class of floral organs, the sterile staminodia, which are similarly transformed into carpels in these flowers. These flowers had no more than ten sepals in total, representing the first whorl sepals and the transformed second whorl organs (Table 1). Surprisingly, we also recovered 25 flowers that presented novel phenotypes due to sterilization of the outer reproductive whorls. In these flowers, there were commonly extra whorls of sepals that appear to be in place of the outer whorls of fertile organs (Fig. 2d, g, i, m; Table 1). Consistent with the variable nature of VIGS, this transformation was incomplete in some cases, yielding sepal/carpel (Fig. 1e–h), petal/carpel (Fig. 1j–l) or sepal/petal (Fig. 1m) chimeras. In all of these flowers, the sepals show various degrees of color loss, regardless of their position in the flower (Fig. 1c–e, g, i, m). These sepals are not white as in *AqANS*-VIGS flowers, but rather pale green (compare Fig. 1b–d, g, or i).

**Table 1:** Organ counts in control and silenced flowers

	Sepals	Petals	Stamens	Staminodia	Carpels
TRV2-*AqANS*	5 ± 0	5 ± 0	43.1 ± 5.8	10 ± 0.97	5 ± 0.51
TRV2-*AqPI*					
Class I	9.5 ± 0.48	0	9.2 ± 4.27	0	55.2 ± 7.95
Class II	21.2 ± 8.79	1 ± 1.8	10.25 ± 18.5	1 ± 4.4	33.5 ± 17.06

### Down-regulation of *AqPI* results in contraction of the *AqAG1* expression domain and the anthocyanin production pathway

We used qRT-PCR to quantify silencing of *AqPI* in all affected floral organs (Fig. 2a). Every tissue tested showed 80–95% silencing except the stamen/petal chimeras, which is not surprising given their retained petal identity. We also examined the expression of the three main *AP3* homologs (Fig. 2b). In the first whorl sepals, we only examined *AqAP3*-*1* and *AqAP3*-*2* because *AqAP3*-*3* is expressed at extremely low levels in these organs [13]. *AqAP3*-*1* expression was slightly lower in the sepals and transformed stamens, but increased in the transformed second whorl petals. We have previously observed increased *AqAP3*-*1* expression in *AqPI*-VIGS tissue [13], so this is not particularly surprising. *AqAP3*-*2* and *AqAP3*-*3* expression was generally decreased in all the organs, except for an increase of *AqAP3*-*3* in the stamen to petal transformed organs, which is consistent with the petal-specific expression of this paralog [13].

In *Aquilegia*, there are two homologs of the C class gene *AGAMOUS*, *AqAG1* and *AqAG2*. In the mature organs used to assess expression in VIGS experiments, *AqAG1* is expressed in both stamens and carpels, but *AqAG2* is only detectable in carpels [13], making it unsuitable for analysis of stamen transformations. Examination of *AqAG1* revealed that its expression was, in fact, dramatically reduced in the sterilized organs (Fig. 2c); however, when stamens were transformed into carpels, *AqAG1* expression remained strong.

Finally, we tested the expression of three members of the anthocyanin synthesis pathway: *ANTHOCYANIDIN SYNTHASE* (*AqANS*), *FLAVONOID 3*-*HYDROXYLASE* (*AqF3H*), and *DIHYDROFLAVONOL 4*-*REDUCTASE* (*AqDFR*) (Fig. 2d). Note that *AqF3H* and *AqDFR* would be expected to be upstream in the anthocyanin synthesis pathway relative to *AqANS* [14]. When *AqANS* is silenced alone, only this member of the synthesis pathway decreases; however, when *AqPI* is silenced alone, all three synthesis genes show decreased expression compared to untreated sepals, although the decrease in *AqF3H* expression was too variable to be significant.

## Conclusions

The primary goal of this experiment was to test whether the B class genes in *Aquilegia* contribute to color production in the petaloid sepals, which required conducting *AqPI* silencing without the presence of the marker *AqANS*. Our results reveal that the B gene homologs do, in fact, promote activation of the anthocyanin synthesis pathway in sepals, but we also recovered unexpected evidence for a role of the B class genes in maintaining the outer extent of the C class domain.

### Homologous transference of function without homeosis in *Aquilegia* sepals

It is very common across the order Ranunculales to observe petaloid sepals, an example of what is termed transference of function, which Baum and Donoghue [15] defined as when “an ecological function performed by one structure comes to be carried out by a new structure.” In this case, the ecological function is pollinator attraction, a role that is often shared between the sepals and petals of these taxa. Unlike the undifferentiated petaloid perianths of the monocots, however, the Ranunculales have morphologically distinct sepals and petals (reviewed [16]). In fact, ranunculid petals, which typically bear nectaries, are often significantly reduced and even entirely lost in some genera. We have hypothesized that this reduction or loss of petals is facilitated by the fact that the sepals have acquired pollinator attractive functions, thereby releasing constraints on the morphology and even the presence of the petals [16].

But how did this transference of function occur? What is the genetic basis of petaloidy in the sepals? Baum and Donoghue posit two general mechanisms for transference of function, one relying on a spatial shift in the expression of a preexisting developmental program, which often results in homeosis, and the other involving convergent evolution of the functional traits without homeosis [15]. As discussed above, several examples of the latter have been observed in which petaloidy has evolved without the apparent involvement of the B class genes. Our initial studies suggested that this was also the case in *Aquilegia*: Although expression of B gene homologs can be detected in late-stage sepals [17], no expression was observed during the stages associated with establishment of organ identity and knockdown of the critical B gene *AqPI* had no effect on sepal morphology or cell types [13].

The current study has demonstrated that the reality of the situation is more complicated. Although the observed late B gene expression does not appear to be required for organ identity, it is important to the production of color in these organs. This indicates that there is what we would term partial process homology between the sepals and petals—the organs share some aspects of their developmental programs. Within Baum and Donoghue’s scheme, this condition would be considered homologous transference of function (utilization of a preexisting genetic program) *without* homeosis. This is distinct from other recently described cases of petaloidy in first whorl organs. In the liliaceous monocot *Tricyrtis*, the B gene homologs promote identity of both perianth whorls [18], while in orchids, it appears that duplications in the *AP3* homologs allow the flowers to differentially encode three distinct petaloid organ identities [11, 12].

The loci that control the fundamental identity of *Aquilegia* sepals, including the production of petaloid-associated papillated cells on the epidermis, remain unknown, but in another ranunculid model, *Nigella damascena*, an *AGL6* homolog has been shown to promote sepal identity [19]. Although the B genes may not be influencing identity per se, it is also interesting to ask if they are solely promoting anthocyanin or perhaps playing additional roles. Studies in *A. thaliana* have found that the B genes directly target loci involved in photosynthesis in order to repress these genes and allow the white color of the petal [20]. We tested whether homologs of these genes, called *BANQUO1/BANQUO2,* were up-regulated in our *AqPI*-VIGS sepals, but we did not recover any consistent results (data not shown). It remains possible that the *Aquilegia* B genes are important for both activation of the anthocyanin pathway and down-regulation of photosynthesis in the sepals.

### Requirement of B gene function for maintenance of C gene expression

In the canonical ABC model, A and C class genes cross-regulate each other, but the B class genes are not essential for the expression of the other two classes [21]. ChIP-seq studies have shown that AP3-containing MADS complexes do target *AG*, which is also known to feedback positively onto its own expression (reviewed [22]). In *A. thaliana*, this positive feedback can be mediated by any MADS complex containing AG and does not strictly require AP3/PI [23]. In two deeply divergent members of the Ranunculales, it appears that the outer extent of the C expression domain requires B gene expression [24]. In *Eschscholzia californica* of the Papaveraceae, the *seirena* mutant, a loss of function allele of a *PI/GLO* homolog, shows the typical transformation of petals into sepals and most stamens into carpels, but the outer stamens are instead transformed into sepals [25]. The authors clearly demonstrate that this is due to homeosis associated with reduced expression of the C class *EscAG1* locus. In *N. damascena*, the situation is somewhat more complicated. Here, an insertional loss of function allele of the *AP3* homolog *NgsAP3*-*3* appears to show transformation of petals into sepals and outer stamens into sepals [26, 27]. This is unexpected because *AP3*-*3* orthologs in the Ranunculaceae are commonly petal specific [16], which makes it surprising that the locus would have an impact on *AG* expression in the stamens. However, in *N. damascena*, the early expression of *NgsAP3*-*3* is broader than just the second whorl, clearly encompassing a region that would cover the outer whorls of stamens [26, 27]. Moreover, the other *AP3* paralogs in *N. damascena* are weakly expressed at very early stages, so *NgsAP3*-*3* is likely to be the predominant *AP3* copy during stamen initiation. Further analysis of the *NgsAP3*-*3* down-regulation phenotype clearly demonstrated that it involves stamen to sepal transformation in conjunction with a contraction in the *NgsAG1* expression domain [19].

Our *A. coerulea* results are, nonetheless, rather surprising because we had not previously observed this phenotype in our TRV2-*AqPI*-*AqANS* study in the Old World species *A. vulgaris* [13]. Perhaps, this was an experimental artifact reflecting the fact that although we recovered many silenced flowers in the original study, they were derived from a small number of plants. Despite the fact that both experiments used VIGS, it may be that the silencing obtained in the current study is stronger or was established earlier in development. Alternatively, there may be differences between species of *Aquilegia* in terms of the degree to which B genes are absolutely necessary for this function. Previous studies of Old World and New World species of *Aquilegia* have uncovered significant differences in the genetics underlying aspects of their floral development [28, 29]. Likewise, in the Papaveraceae, the phenotype was observed in *Eschscholzia,* but not when B class homologs were silenced in *Papaver* [30]. In any case, we now appear to have independent sets of data from three divergent ranunculid taxa, demonstrating that B gene homologs are important for the initiation and/or maintenance of the outer extent of the *AG* homolog expression domain.

There are a number of possible contributors to the dependence of C gene expression on B gene function. First, as already noted, *Arabidopsis thaliana* MADS complexes containing AP3/PI do participate in positive feedback onto *AG*, so it may be that the process of developmental system drift [31] has produced a different pattern of regulatory redundancies that increased the importance of the B genes in this process. It is also interesting to note that in all three of these taxa—*Eschscholzia, Nigella, and Aquilegia*—the stamens are not present as a single whorl, but rather a broad domain encompassing many organs. In *A. thaliana*, *AG* expression is promoted by the activity of LEAFY (LFY) in conjunction with several other factors, including WUSCHEL (WUS), which is tightly expressed in the center of the meristem, but whose protein diffuses non-cell autonomously outward [32]. We currently know nothing about how *AG* homolog expression is activated in Ranunculales or in any angiosperm with a broad domain of stamens. Is activation still dependent primarily on WUS? Does WUS diffuse all the way to the outermost extent of the stamen domain? Perhaps not. It may be that spatial limitations in the extent of WUS diffusion have led to the evolution of a greater dependence on the B class genes for activation of *AG* in the outer regions of the stamen domain. Further genetic dissection of ABC gene regulation in multiple ranunculid models will provide insight into the developmental basis of this common floral morphology.

## Methods

### Virus-induced gene silencing

The *Aquilegia* VIGS protocol and construction of the TRV2-*AqANS* positive control plasmid have been previously described [13, 33]. To make the TRV2-*AqPI* construct, we PCR-amplified a 326-bp fragment of *AqPI* using primers that added XbaI and BamHi sites to the respective 5′ and 3′ ends of the PCR product (5′ GGTCTAGAGCTTGGCGGGAATGATAGAGAAATGGAAAATG, 5′ AAGGATCCCCATAATCAAGAGAAACTTTAAAATCATGGATA). This PCR product was used to produce the TRV2-*AqPI* construct in a manner similar to Gould and Kramer [33]. A total of 90 *A. coerulea* “Origami” plants at the four to six true leaf stage were vernalized at 4°C for three weeks, and then, one day after the plants had been removed from vernalization, they were treated as described for seedlings in [33]. Totally, 90 control plants were also treated with TRV2-*AqANS*. Flowers showing any floral phenotypes were photo-documented and, upon maturation, the flowers were dissected. All individual perianth organs were photographed using a Kontron Elektronik ProgRes 3012 digital camera mounted on a Leica WILD M10 dissecting microscope (Harvard Imaging Center). For the purposes of counting organ types, organs were scored as sepals if they lacked spurs and had lanceolate apices; organs were scored as petals if they had rounded apices and some degree of spur development; organs were scored as stamens if they had filaments and some degree of anther development; organs were scored as staminodia if they were sterile; and organs were scored as carpels if they possessed ovules. For every flower showing silencing, a selection of organs from each whorl was either flash frozen at − 80° for subsequent RNA analysis or fixed in freshly prepared, ice-cold FAA for scanning electron microscopy (SEM) analysis. This process was also repeated for several untreated flowers as well as flowers that were treated with TRV2-*AqANS* as controls. SEM analysis and light microscopy were performed as described in [13].

### Expression analysis of VIGS-treated organs

Total RNAs were prepared from collected floral organs using the Qiagen RNeasy kit according to manufacturer’s instructions (Qiagen, Valencia, CA, USA). Each RNA sample was treated with TURBO DNase (Ambion by Life Technologies, Carlsbad, CA, USA) following manufacturer’s instructions. Individual cDNAs were synthesized using 1 µg of total RNA (SuperScript III™ First Strand Synthesis, Invitrogen, San Diego, CA, USA). All primer sequences are listed in Additional file 1: Table S1. The primers were tested for amplification efficiency using a cDNA dilution series as templates. The efficiency (*E*) of the primers was determined using the slope of the linear regression line in Microsoft Excel 2015 (*E* = 10^[(−1/slope)−1]^ × 100). The specificity of each primer pair was verified by dissociation curve analysis (60–95 °C). The quantitative RT-PCR was carried out using PerfeCTa qPCR FastMix, Low ROX (Quanta Biosciences Inc., Gaithersburg, MD, USA) in the Stratagene Mx3005P QPCR system (Agilent, Santa Clara, CA, USA). Relative expression levels were calculated based on the 2 − ΔΔ*Ct* method [34]. *AqIPP2* (GenBank KC854337) was used as the reference gene for expression normalization.

Several different sample types were examined. We determined *AqPI* expression in five different classes of tissue selected to represent a range of phenotypes: first whorl organs (sepals showing color changes), second whorl organs (petal to sepal transformations), and three classes of stamen transformations (stamen to sepal, stamen to petal, and stamen to carpel). Each class contained three to six separate samples, and each sample was analyzed in three technical replicates. These values were averaged across all of the silenced organs in that class and compared to equivalent pooled RNA samples from organs dissected from *AqANS*-VIGS flowers (Fig. 2a). The silenced sepals, petal to sepal transformants, stamen to sepal transformants, and stamen to petal transformants were similarly tested for the expression of all three main *AqAP3* paralogs (Fig. 2b). These experimental values were normalized to *AqAP3*-*1, AqAP3*-*2,* and *AqAP3*-*3* expression levels in control *AqANS*-VIGS samples. We examined *AqAG1* expression in a variety of transformed stamen classes and normalized those values to *AqAG1* expression in *AqANS*-VIGS stamens (Fig. 2c). Lastly, we determined the expression of three members of the anthocyanin synthesis pathway (*AqANS*, *AqF3H,* and *AqDFR*) in sepals from *AqANS*-VIGS flowers and *AqPI*-VIGS flowers, with the values normalized to the corresponding gene expression levels of those genes in wild-type sepals. All results presented are the mean ± standard deviation of the examined samples, normalized to the appropriate reference sample as described above. All error bars represent standard deviation, and unpaired Student’s *t*-tests were used to determine the statistical significance of differences between experimental and control values.

## Additional file

**Additional file 1: Table S1.** Primer sequences.

## Abbreviations

*AP3*: *APETALA3*
*PI*: *PISTILLATA*
*ANS*: *ANTHOCYANIDIN SYNTHASE*
*AG*: *AGAMOUS*
VIGS: virus-induced gene silencing
*F3H*: *FLAVONOID 3*-*HYDROXYLASE*
*DFR*: *DIHYDROFLAVONOL 4*-*REDUCTASE*
*E. californica*: *Eschscholzia californica*
*N. damascena*: *Nigella damascena*
LFY: LEAFY
WUS: WUSCHEL
