# Supplementary material for: Aquilegia B gene homologs promote petaloidy of the sepals and maintenance of the C domain boundary
Source: EvoDevo. 2017 Nov 28;8:22. doi: 10.1186/s13227-017-0085-7 (PMC5704387; doi:10.1186/s13227-017-0085-7)
Supplement: Supplementary file 1 — Additional file 1: Table S1. Primer sequences. [file 13227_2017_85_MOESM1_ESM.docx]

**Table S1.** Primer sequences

| **qRT-PCR Primers** | |
| --- | --- |
| **Locus** | **Primer Sequences** |
| *AqAP3-1* | F 5’ GCCTTGAAGAAGCAGAAAGAGACC  R 5’ GAGTAAACTTTCTTTGTGCAACAATCTTT |
| *AqAP3-2* | F 5’ CATGAGTATATTAGTCCTTCTTGCACACAC  R 5’ GGAAGCTCAATTCATCCAGATCTTCT |
| *AqAP3-3* | F 5’ CTGAGTTTATTAGTCCTTCTACCACAACG  R 5’ GTCCCGAACAAGTTTCACAGACTC |
| *AqPI* | F 5’ AGAAGCTGTGGGATGCAAAGC  R 5’ GGGCTTCTTCAATAGGAATGAGTTC |
| *AqAG1* | F 5’ GCACAGTTTTACCAGCAAGAAGTGA  R 5’ ACTCTCCCACCAATTTCCTGTTATG |
| *AqANS* | F 5’ AGTTTGCTAATGATGTGGCGTCA  R 5’ GCTGTTACCTCCGTGTATTCAGTAGG |
| *AqF3H* | 5’ TTAATGCTTGTGAAGACTGGGGAAT  5’ CCAGTCCTGCACTGCCTCTCC |
| *AqDFR* | 5’ GCCACCATCATGGATGTTGCA  5’ TGAAGCAACAGCCTCAATGAACA |
| *AqIPP2* | F 5’ CAGGTGAAGACGGACTGAAGTTATC  R 5’ CCAAGACTGGAAAAAAGACCACAC |
